# Aberrant differentiation of epithelial progenitors is accompanied by a hypoxic microenvironment in the paraquat-injured human lung

**DOI:** 10.1038/s41421-023-00598-0

**Published:** 2023-09-26

**Authors:** Yanxiao Wang, Ennan Bin, Jie Yuan, Man Huang, Jingyu Chen, Nan Tang

**Affiliations:** 1 National Institute of Biological Sciences, Beijing, China; 2 Graduate School of Peking Union Medical College, Beijing, China; 3 Center for Lung Transplantation, Second Affiliated Hospital, Zhejiang University School of Medicine, Hangzhou, Zhejiang, China; 4 Wuxi Lung Transplantation Center, Wuxi People’s Hospital affiliated to Nanjing Medical University, Wuxi, Jiangsu, China; 5 Tsinghua Institute of Multidisciplinary Biomedical Research, Tsinghua University, Beijing, China

**Keywords:** Regeneration, Stem-cell differentiation

Dear Editor,

Paraquat is a toxic herbicide that can cause severe lung injury, leading to alveolar epithelial cell death, subsequent lung fibrosis and respiratory failure. Understanding repair processes following paraquat injury is critical for developing potential therapeutic strategies to treat paraquat-poisoned patients. However, the repair program in paraquat-injured lungs is currently unknown. In this study, we analyzed lung parenchyma samples from an 18-year-old female patient who had ingested paraquat and subsequently underwent a double-lung transplantation on the 34th day after poisoning^1^.

Histological analyses revealed that alveolar structure was severely disrupted by paraquat toxicity. Immunostaining experiments demonstrated the absence of PDPN^+^ alveolar type 1 (AT1) epithelial cells and a dramatic decrease in proSPC^+^ alveolar type 2 (AT2) epithelial cells in the paraquat-injured lung (Fig. 1a; Supplementary Fig. S1a). Instead, these areas were filled with α-SMA^+^ myofibroblasts (Fig. 1a). The surviving proSPC^+^ AT2 cells were predominately clustered at the periphery of the paraquat-injured lung, with a few scattered around the distal airway (Supplementary Fig. S1a, b). To investigate repair programs in the severely injured lung, we conducted single-cell RNA sequencing (scRNA-seq) analyses. Following an integrated quality control pipeline, we obtained a comprehensive collection of 19,171 transcriptomes, which were annotated into four broad cell type groups: *EPCAM*^+^ epithelial cells (*n* = 4120), *DCN*^+^ stromal cells (*n* = 2491), *PTPRC*^+^ immune cells (*n* = 10,431), and *CDH5*^+^ endothelial cells (*n* = 2129) (Supplementary Fig. S2a). Based on canonical lineage markers and distinct molecular signatures of individual cell types, we identified a total of 45 cell types/states in the paraquat-injured lung (Supplementary Fig. S2b–e and Tables S1, S2).

**Fig. 1:**
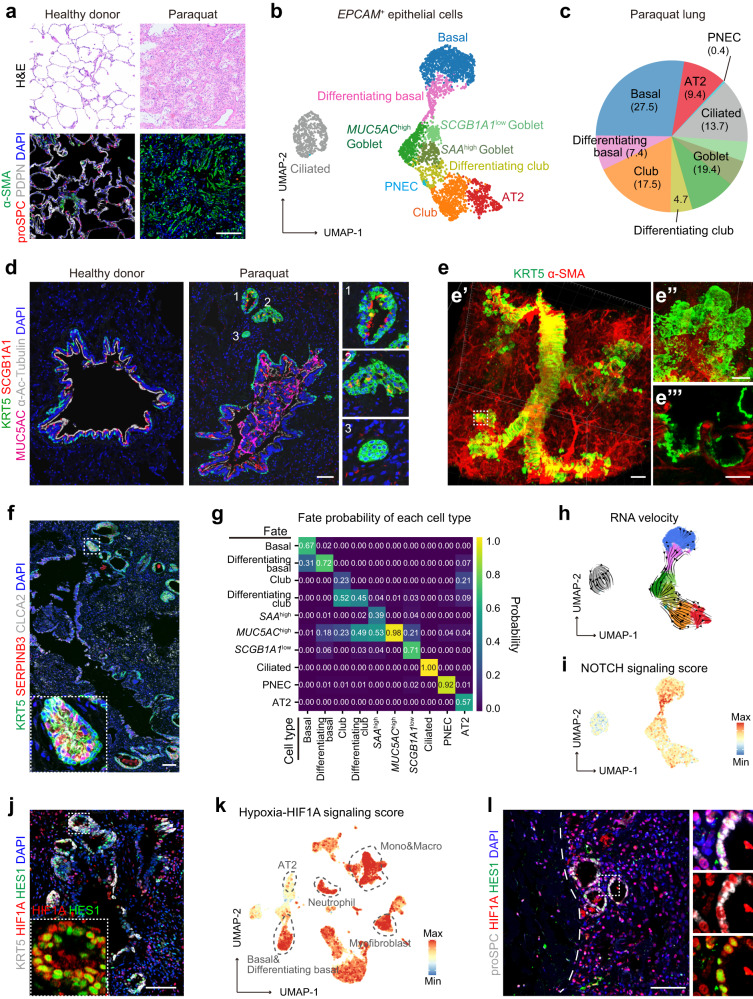
Aberrant epithelial repair programs in the paraquat-injured human lung. **a** Hematoxylin and eosin (H&E) analysis (top panel) and immunostaining (bottom panel) for proSPC (AT2 cell marker), PDPN (AT1 cell marker), and α-SMA (myofibroblast marker) of a healthy lung and a paraquat-injured lung. Scale bar, 100 μm. **b** Uniform Manifold Approximation and Projection (UMAP) embedding of epithelial cells in the paraquat-injured lung and subpopulation annotation. **c** Relative proportions of each epithelial cell type derived from **b**. **d** Immunostaining for KRT5 (basal cell marker), SCGB1A1 (club and goblet cell marker), MUC5AC (goblet cell marker), and α-Ac-Tubulin (ciliated cell marker) of the healthy lung and the paraquat-injured lung. The honeycomb pods around the distal airway of the paraquat-injured lung were magnified on the right panels. Scale bar, 100 μm. **e** Wholemount staining of the paraquat-injured lung using antibodies against KRT5 and α-SMA. The magnified wholemount honeycomb pod structures are shown in **e″**. The Z section in **e‴** shows that KRT5^+^ cells in the pods are connected with KRT5^+^ basal cells in airways. Scale bar in **e′**, 500 μm; Scale bars in **e″** and **e‴**, 100 μm. **f** Immunostaining of the paraquat-injured lung using antibodies against KRT5, SERPINB3, and CLCA2 (differentiating basal cell markers). The inset in the lower left corner shows a zoomed image of the white dotted box region. Scale bar, 100 μm. **g** CellRank’s coarse-grained and directed transition of epithelial cells in the paraquat-injured lung. Heatmap represents the mean absorption probabilities of every cell in the given cell types to the terminal fates. **h** Pseudotime trajectory analysis calculated from UMAP embedding of epithelial cells in **b** by RNA velocity. Arrows indicate predicted lineage trajectories. **i** NOTCH signaling score in *EPCAM*^+^ epithelial cells of the paraquat-injured lung projected onto UMAP embedding in **b**. **j** Immunostaining of the paraquat-injured lung using antibodies against KRT5, HIF1A, and HES1. The inset in the lower left corner shows a zoomed image of the white dotted box region. Scale bar, 100 μm. **k** Hypoxia-HIF1A signaling score in all cells of the paraquat-injured lung projected onto the UMAP embedding in Supplementary Fig. S2b. **l** Immunostaining of the paraquat-injured lung using antibodies against proSPC, HIF1A, and HES1. The white dotted line indicates the periphery of the lung. Scale bar, 100 μm.

Unbiased clustering analysis of *EPCAM*^+^ epithelial cells revealed ten distinct subpopulations (Fig. 1b). Consistent with the immunostaining results, there was no *PDPN*^+^ AT1 cell population, indicating a complete loss of gas-exchange units in the paraquat-injured lung. These ten subpopulations included *SFTPC*^+^ AT2 cells and nine subpopulations of airway epithelial cells, the latter of which comprise seven classically defined cell types (basal, *MUC5AC*^high^ goblet, *SAA*^high^ goblet, *SCGB1A1*^low^ goblet, club, ciliated, and pulmonary neuroendocrine cells) and two intermediate states (differentiating basal cells and differentiating club cells) (Fig. 1b; Supplementary Fig. S3a). Differentiating basal cells expressed both basal cell markers (*KRT5* and *KRT15*) and goblet cell markers (*SERPINB3*, *SERPINB4*, and *CLCA2*)^2^ (Supplementary Fig. S3a), indicating that they are progenitors of goblet cells. Newly identified differentiating club cells expressed markers of both club cells (*SCGB3A2* and *SCGB1A1*) and goblet cells (*MUC5B*) (Supplementary Fig. S3a). Given that goblet cells are recognized as a terminally differentiated cell type, the differentiating club cells may represent an intermediate stage of club cell to goblet cell differentiation.

In healthy donor lungs, *EPCAM*^+^ epithelial cells consist of both alveolar epithelial cells (AT1 and AT2 cells) and airway epithelial cells, with alveolar epithelial cells accounting for over 40% of the *EPCAM*^+^ population^3,4^. However, in the paraquat-injured lung, the proportion of alveolar epithelial cells (the AT2 cell population) significantly decreased to 9.4%, while the proportion of airway epithelial cells increased to 90.6% (Fig. 1c). Specifically, the proportions of airway stem/progenitor cells, including basal cells (27.4%), club cells (17.5%), differentiating basal cells (7.4%), and differentiating club cells (4.7%), were significantly increased (Fig. 1c). These findings suggest that the paraquat-injured lung undergoes an active airway repair program but an inadequate alveolar repair program.

To investigate distribution of the increased airway epithelial cells in the paraquat-injured lung, we performed immunostaining analyses. Similar to the healthy lung, the pseudostratified epithelial cells of distal airways in the paraquat-injured lung consisted of KRT5^+^ basal cells, SCGB1A1^+^MUB5AC^–^ club cells, and α-Ac-Tubulin^+^ ciliated cells (Fig. 1d). However, we observed mucus accumulations and desquamated epithelial cells obstructing airway lumen in the paraquat-injured lung. Furthermore, we observed many “honeycomb pod” structures located in alveolar regions around distal airways, which were not observed in the healthy lung (Fig. 1d). The predominant epithelial cells in honeycomb pods were positive for KRT5 (Fig. 1d). By wholemount staining, we further found that these KRT5^+^ honeycomb epithelial cells still maintained a close connection with the KRT5^+^ distal airway epithelial cells (Fig. 1e; Supplementary Videos S1, S2). Most KRT5^+^ epithelial cells in honeycomb pods highly expressed markers of differentiating basal cells, SERPINB3 and CLCA2 (Fig. 1f). These results suggest that airway basal cells in the distal airways give rise to differentiating basal cells and form abnormal honeycomb pods in the paraquat-injured lung.

Previous studies have shown that airway stem/progenitor cells not only replenish lost airway epithelial cells but also participate in ectopic alveolar repair program after lung injuries^5–8^. To further elucidate lineage relationships of airway stem/progenitor cells in the context of paraquat-induced severe lung injury, we employed RNA velocity and CellRank analyses. Our analyses revealed that basal cells possess a higher propensity to give rise to differentiating basal cells (Fig. 1g, h). Club cells can differentiate into goblet cells through the differentiating club cells, supporting the notion that differentiating club cells represent an intermediate stage connecting club cells to goblet cells (Fig. 1g, h). Previous studies utilizing mouse models of lung injury have indicated that airway progenitor cells can differentiate into AT2 and AT1 cells^5–7^. However, our analyses showed limited probabilities of basal cells, differentiating basal cells, and club cells differentiating into AT2 cells in the paraquat-injured lung (Fig. 1g, h). In contrast, AT2 cells in the paraquat-injured lung display a higher propensity of differentiating into club cells. Collectively, these results suggest that both AT2 cells and airway stem/progenitor cells in the paraquat-injured lung exhibit a significantly reduced capacity to regenerate alveolar epithelium, ultimately leading to an irreversible loss of gas-exchange units.

To explore regulatory pathway underlying aberrant repair of airway stem/progenitor cells to alveolar epithelium, we compared gene expression in differentiating basal cells in healthy donor lungs^9^ and the paraquat-injured lung. We found that genes involved in regulation of NOTCH signaling and reactive oxygen species responses are highly upregulated in the paraquat-injured lung (Fig. 1i; Supplementary Fig. S3b). Expression of *HES1* and *HEY1*, target genes of NOTCH signaling, was significantly increased, supporting the activation of NOTCH signaling in differentiating basal cells (Fig. 1i, j; Supplementary Fig. S3c). Given that reactive oxygen species have been implicated in hypoxic response^10^, we further analyzed expression of hypoxia-inducible factor 1 alpha (HIF1A), a well-known hallmark of hypoxia^11^. Immunostaining analysis demonstrated that the majority of honeycomb basal cells were positive for HIF1A (Fig. 1j), indicating the activation of hypoxia-induced signaling in these differentiating basal cells. Notably, HES1 is also highly expressed in most of the HIF1A^+^ honeycomb basal cells (Fig. 1j). These findings are consistent with a previous study reporting that hypoxia can activate NOTCH signaling and block the differentiation of airway progenitor cells into alveolar epithelial cells in H1N1 influenza virus-injured lungs^12^. Taken together, our results indicate that hypoxia-induced activation of NOTCH signaling in differentiating basal cells leads to a diminished capacity for generating alveolar epithelial cells in the paraquat-injured lung.

Significant increase of HIF1A^+^ cells and their widespread distribution in the paraquat-injured lung indicated that lung microenvironment might be extensively hypoxic. Indeed, in addition to differentiating basal cells, other cell types including basal cells, AT2 cells, monocytes, macrophages, neutrophils and myofibroblasts, generally exhibited high hypoxia scores (Fig. 1k). HIF1A^+^ AT2 cells were also observed at the periphery of the paraquat-injured lung, and some AT2 cells co-expressed HES1 (Fig. 1l), suggesting that hypoxia-induced NOTCH signaling was also activated in AT2 cells. Sustained NOTCH signaling activation has been demonstrated to inhibit AT2 to AT1 cell differentiation in mouse lungs^13^. We speculate that the hypoxia-induced activation of NOTCH signaling in AT2 cells may impair differentiation of AT2 to AT1 cells during alveolar repair in this paraquat-injured lung.

In this study, we experimentally demonstrated that epithelial regeneration programs in the paraquat-injured lung are disrupted. Massive cell death after paraquat poisoning leads to significant infiltration of immune cells, loss of gas-exchange units, and lung fibrosis (Supplementary Fig. S2b), resulting in an extensive hypoxic microenvironment in the lung tissues. This microenvironment may contribute to the aberrant repair programs by activating NOTCH signaling and by disrupting differentiation of airway and alveolar stem/progenitor cells, which exacerbates lung tissue damage. Our findings suggest that lung transplantation may be the only viable treatment for patients with severe paraquat poisoning. Importantly, our study highlights the crucial role of an appropriate microenvironment in regenerating functional alveolar units, which depends on differentiation of newly formed AT1 cells. Since hypoxia is a common feature in severe lung diseases, future therapeutic strategies targeting hypoxic lung microenvironment, such as reducing hypoxia or inhibiting NOTCH signaling, may hold promise for treating severe lung injuries.

### Supplementary information

Supplementary informaiton

Video S1

Video S2

Dataset 1

Dataset 2

## Data Availability

scRNA-seq data are deposited at the Gene Expression Omnibus depository (accession number: GSE231647).
