# Supplementary material for: Aberrant differentiation of epithelial progenitors is accompanied by a hypoxic microenvironment in the paraquat-injured human lung
Source: Cell Discov. 2023 Sep 26;9:98. doi: 10.1038/s41421-023-00598-0 (PMC10522573; doi:10.1038/s41421-023-00598-0)
Supplement: Supplementary file 1 — Supplementary informaiton [file 41421_2023_598_MOESM1_ESM.pdf]

Supplementary information for

Aberrant differentiation of epithelial progenitors is accompanied by a hypoxic microenvironment in the paraquat-injured human lung

Yanxiao Wang[1*], Ennan Bin[1*], Jie Yuan[1,2], Man Huang[3], Jingyu Chen[3,4#], Nan Tang[1,5#]

[1]National Institute of Biological Sciences, Beijing 102206, China.

[2]Graduate School of Peking Union Medical College, Beijing 100730, China.

[3]Center for Lung Transplantation, Second Affiliated Hospital, Zhejiang University School of Medicine, Hangzhou 310052, China.

[4]Wuxi Lung Transplantation Center, Wuxi People's Hospital affiliated to Nanjing Medical University, Wuxi 214023, China.

[5]Tsinghua Institute of Multidisciplinary Biomedical Research, Tsinghua University, Beijing 100084, China.

*These authors contributed equally: Yanxiao Wang and Ennan Bin

[#]Correspondence: tangnan@nibs.ac.cn (N.T.), chenjy@wuxiph.com (J.C.)

This file includes:

Supplementary Fig. S1

Supplementary Fig. S2

Supplementary Fig. S3

Titles of Supplementary Table S1, S2

Titles of Supplementary Video S1, S2

Materials and Methods

Supplementary References

Supplementary Fig. S1

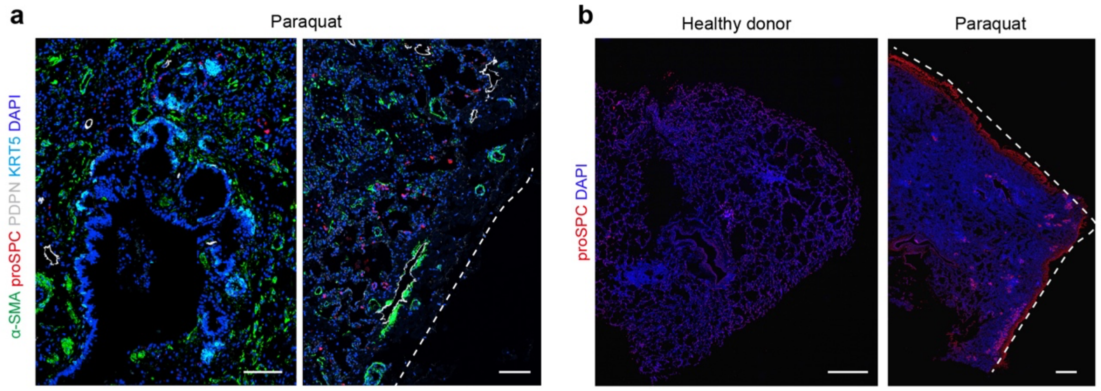

Supplementary Fig. S1: Histological changes of epithelial cells in the paraquat-injured human lung. a Immunostaining results of the paraquat-injured lung using antibodies against proSPC, PDPN, KRT5, and α-SMA. The white dot line indicates the periphery of the lung. Scale bar, 100 μm. **b** Immunostaining for proSPC of a healthy donor lung and a paraquat-injured lung. The white dot line indicates the periphery of the lung. Scale bar, 1 mm.

Supplementary Fig. S2

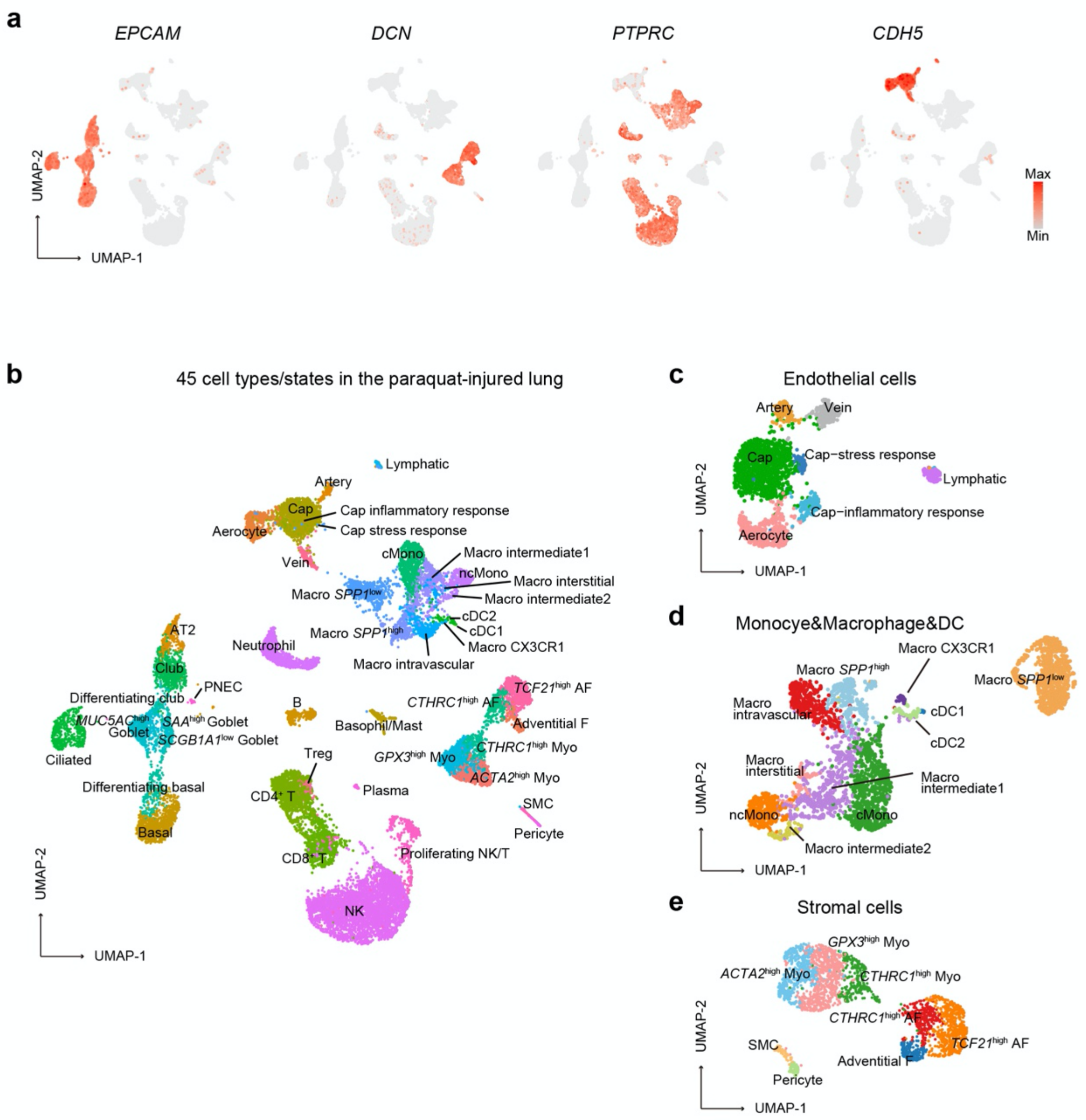

Supplementary Fig. S2: Single-cell RNA sequencing analysis of the paraquat-injured human lung. a UMAP embeddings of 19171 transcriptomes show the expression of cell specific markers genes of the four broad groups in the paraquat-injured human lung. **b-e** UMAP embeddings show the annotation of cell types from all four broad groups (**b**), endothelial cells (**c**), subgroups of immune cells (monocyte, macrophage, and dendritic cell; DC) (**d**), and stromal cells (**e**). In immune cells, we observed increased neutrophils and natural killer cells. In macrophage population, the alveolar macrophages were absent in the paraquat-injured lung. There were two distinct macrophage populations, including *SPP1*^{high} macrophages, *SPP1*^{low}

macrophages. In stromal cells, the number of myofibroblasts (Myo) increased significantly. In addition to an increased number of previously identified *CTHRC1*^{high} Myo and *ACTA2*^{high} Myo subpopulations, a new *GPX3*^{high} Myo subpopulation in the paraquat-injured lung were observed. The *GPX3*^{high} Myo subpopulation is characterized by upregulated genes involved in the response to hypoxia. Macrophage (Macro), classical monocyte (cMono), non-classical monocyte (ncMono), conventional type 1 dendritic cell (cDC1), conventional type 2 dendritic cell (cDC2), natural killer (NK), T regulatory (Treg), alveolar fibroblast (AF), adventitial fibroblast (Adventitial F), myofibroblast (Myo), capillary (Cap), pulmonary neuroendocrine cell (PNEC).

Supplementary Fig. S3

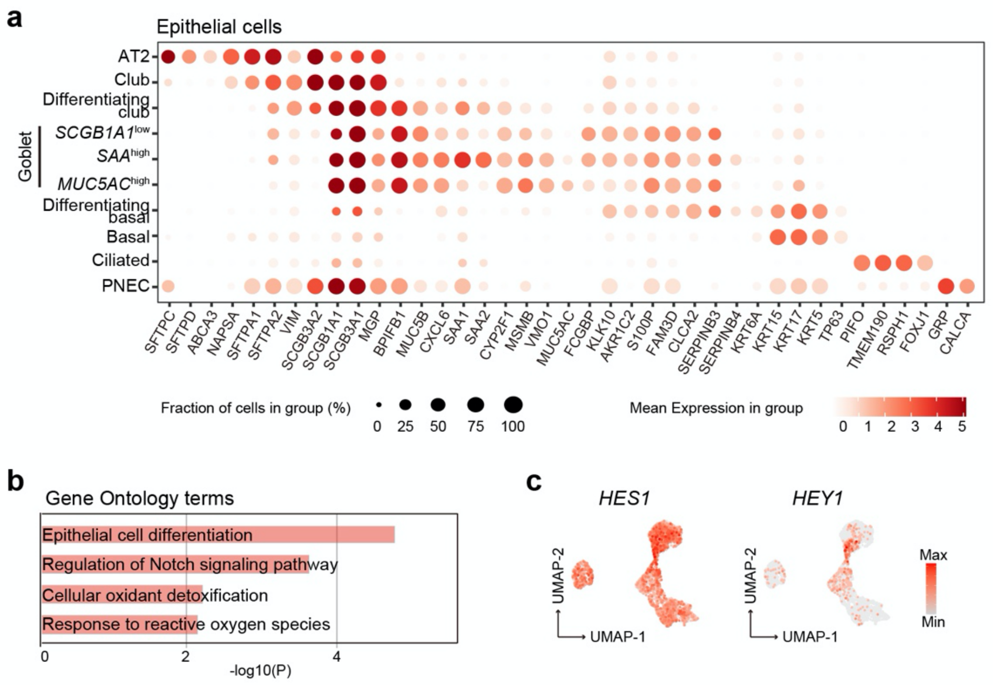

Supplementary Fig. S3: Single cell RNA sequencing analysis of the epithelial cells in the paraquat-injured human lung. a Dot plot of the expression of cell type marker genes in the epithelial cell types. Dot size indicates percentage of cells per cluster with any mRNAs detected, and color shows scores of log-normalized mRNA counts. **b** Gene ontology analysis of the differentially expressed genes of differentiating basal cells in the paraquat-injured lung compared to differentiating basal cells in the healthy donor lung. **c** UMAP plots show the expression of NOTCH signaling targets, *HES1* and *HEY1*, in *EPCAM*$^{+}$ epithelial cells.

Supplementary Table S1: The top 30 marker genes of the 45 cell types/states in the paraquat-injured human lung.

Supplementary Table S2: Differentially expressed genes upregulated in individual cell types of the paraquat-injured human lung.

Supplementary Video S1 and S2: 3D reconstruction of the wholemount stained lung tissue of paraquat-injured lung. Wholemount immunostaining for KRT5 (green) and α-SMA (red) of the paraquat-injured lung tissue. Most of the honeycomb pods are located in alveolar regions around distal airways and maintain a close connection with $KRT5^+$ distal airway epithelial cells. **S1,** horizontal rotation**. S2,** vertical rotation**.** Scale bar, 500 μm.

Materials and Methods

Histology and immunostaining assay

Human lung tissues were fixed in 4% paraformaldehyde (PFA) for 24 hours at 4°C. After dehydration, the tissues were embedded in paraffin. 5 μm sections of lung tissues were placed on the coated slides. After deparaffinization and rehydration, the sections were used for the following hematoxylin and eosin (H&E) and immunostaining assay.

For H&E staining, the nucleus was stained by hematoxylin (ab150678, Abcam) for 3 minutes (mins) and the cytoplasm was stained by eosin (HT110280, Sigma-Aldrich) for 1 min. After dehydration and clearing steps, slices were sealed with neutral resin. The slides were scanned by Olympus VS120.

For immunostaining assay, slices were incubated with 3% H_2O_2 to neutralize endogenous peroxidase activity. Antigen unmasking was performed using heat treatment with citrate solution (pH 6.0), followed by blocking of nonspecific binding sites with goat serum for 1 hour at 37°C. Then the slices were incubated overnight at 4°C with the primary antibodies against proSPC (Abcam, ab40879, 1:1000), PDPN (Abcam, ab109059, 1:1000), α-SMA (Cell signal technology, 19245, 1:300), SCGB1A1 (Millipore, 07-623, 1:2000), MUC5AC (Abcam, ab3649, 1:500), α-Tubulin (Sigma, T6793, 1:1000), Keratin5/KRT5 (Abcam, ab52635, 1:1000), Serpinb3 (Abcam, ab154971, 1:200), CLCA2 (Proteintech, 19273-1-AP, 1: 200), HIF1A (Novus, NB100-479, 1:100), HES1 (Cell signal technology, 11988, 1:300). The secondary antibodies (Beijing Zhongshan Golden Bridge Biotechnology Co.) were added and then incubated at room temperature for 30 mins before the TSA reaction. DAPI was used to highlight nuclei. The images were captured by Zeiss LSM 980.

iDISCO-based whole mount staining

The iDISCO protocol was followed as previously described.[1] Briefly, lung tissue of the

paraquat-injured lung (about 8 x 5 x 4 mm^3) was fixed in 4% PFA for 72 hours at 4°C and then was permeabilized by a MeOH pre-treatment step. After blocking, sample was immunolabeled with 1.5 mL of primary antibody against KRT5 (Abcam, ab52635, 1:300) and Cy3-conjugated anti-α-SMA (Sigma-Aldrich, 1:300, C6198) for 7 days at 37°C. After 1 day of washing at room temperature (RT), sample was immunolabeled with 1.5 mL of secondary antibody mixture for 5 days at 37°C. After stepwise dehydration, sample was cleared with dichloromethane (DCM) (Sigma-Aldrich 270997) and dibenzyl ether (DBE) (Sigma-Aldrich 108014). The images were captured by Zeiss light sheet microscope. Imaris imaging software was employed to reconstruct the wholemount stained lung tissue.

Sample preparation for single-cell RNA sequencing analysis

Lung specimens of the paraquat poisoning patient were obtained from the lung removed at the time of lung transplantation. For primary cell isolation, the lung specimens without mesothelial layer were minced mechanically into small pieces (<1 mm^3) and digested in modified DMEM medium with an enzyme cocktail containing 10 U/mL neutral protease (Worthington, LS02111), 400 U/mL collagenase type I (GIBCO, 17100-017), 8 U/mL elastase (Worthington, 2294), and 0.66 U/mL DNase I (Roche, 10104159001) for 50 mins at 37°C. To stop the enzymatic reaction, the digested tissues were added 10% FBS (Gibco, 1767839) and incubated for 10 mins at RT. Digested tissue was filtered using 100 μm- and 40 μm-cell strainer. The filtrated suspension was centrifuged at 400 g at 4°C for 8 mins to collect the dissociated cells. The cell pellet was resuspended in RBC lysis buffer and incubated for 2 mins at RT to lyse the red blood cells. After centrifugation, the cell pellet was resuspended in freezing DMEM+5% FBS to stain with PE anti-human EPCAM (1:400, 324205, Biolegend), APC anti-human CD31 (1:400, 303115, Biolegend), FITC anti-human CD45 (1:400, 304017, Biolegend) at 4 °C for 30 mins. Then the cell suspension was centrifuged at 400 g at 4°C for 5 mins. The cell pellet was resuspended with DMEM+2% FBS and filtered

through a 40 μm cell strainer again before the sorting process. DAPI was used to label dead cells. Sorting was performed on a BD FACS Aria III appliance.

After centrifugation at 400 g at 4°C for 5 mins, sorted $EPCAM^{+}CD31^{-}CD45^{-}$ cells (epithelial cells), $EPCAM^{-}CD31^{+}CD45^{-}$ cells (endothelial cells), $EPCAM^{-}CD31^{-}CD45^{+}$ cells (immune cells), and $EPCAM^{-}CD31^{-}CD45^{-}$ cells (stromal cells), were separately resuspended in 1 mL of freezing PBS+0.04 % BSA. For cell concentrations and viability, cells were stained with Trypan blue and then counted on hemocytometer.

Single-cell RNA sequencing analysis

Sorted lung $EPCAM^{+}CD31^{-}CD45^{-}$ cells, $EPCAM^{-}CD31^{+}CD45^{-}$ cells, $EPCAM^{-}CD31^{-}CD45^{+}$ cells, and $EPCAM^{-}CD31^{-}CD45^{-}$ cells harvested by FACS were processed following 10x genomics protocol to construct libraries. The four libraries were sequenced using Illumina Hiseq X10. CellRanger Count v3.1 (10x Genomics) was used to align reads onto GRCh38 reference genome. Primary scRNA-seq data are deposited at the GEO depository: GSE231647.

Seurat package (v4.0.1) was used to perform dimensionality reduction, clustering, and visualization for the scRNA-seq data presented using UMAP plots. To filter valid cell barcodes, cells with more than 6000 genes profiled or more than 20% of the transcriptome of mitochondrial origin were then removed from each object. To limit the bias in gating of FACS, the four Seurat objects were then combined into a merged dataset. According to the general markers *EPCAM*+ (epithelial cells), *CDH5*+ (endothelial cells), *PTPRC*+ (immune cells), *DCN*+ (stromal cells), the cells of the paraquat-injured lung were separated into four main groups. The cell type annotation depended on the differentially expressed genes (DEGs) of each individual cell type and the previously reported lineage markers[2-4]. To identify the DEGs between cell types in each cell type, the “FindMarkers” implemented in the Seurat was used by comparing each individual cell type to all other cells within the major cell type cluster

(log_2-transformed fold change > 0.2). The “AddModuleScore” function was used to calculate the average expression levels of genes in NOTCH signaling pathway[5] and hypoxia-HIF1A signaling pathway[6,7] on a single-cell level.

Trajectory analysis

The CellRank algorithm was used to explore the cell state dynamics within the epithelial cells.[8] Multiple macrostates per cell type were set manually to combine into one terminal cell state of each cell type. After recovered the velocity information[9] on the embeddings, the CellRank was performed by setting the kernel with weighing the velocity kernel 0.8 and the connectivity kernel 0.2.

Ethics approval

This study was approved by the Ethics Committees of Nanjing Medical University (2020-374).

Supplementary References

1 Renier, N. *et al.* iDISCO: a simple, rapid method to immunolabel large tissue samples for volume imaging. *Cell* **159**, 896-910, doi:10.1016/j.cell.2014.10.010 (2014).

2 Adams, T. S. *et al.* Single-cell RNA-seq reveals ectopic and aberrant lung-resident cell populations in idiopathic pulmonary fibrosis. *Sci Adv* **6**, eaba1983, doi:10.1126/sciadv.aba1983 (2020).

3 Madissoon, E. *et al.* A spatially resolved atlas of the human lung characterizes a gland-associated immune niche. *Nat Genet* **55**, 66-77, doi:10.1038/s41588-022-01243-4 (2023).

4 Travaglini, K. J. *et al.* A molecular cell atlas of the human lung from single-cell RNA sequencing. *Nature* **587**, 619-625, doi:10.1038/s41586-020-2922-4 (2020).

5 Xi, Y. *et al.* Local lung hypoxia determines epithelial fate decisions during alveolar regeneration. *Nat Cell Biol* **19**, 904-914, doi:10.1038/ncb3580 (2017).

6 Benita, Y. *et al.* An integrative genomics approach identifies Hypoxia Inducible Factor-1 (HIF-1)-target genes that form the core response to hypoxia. *Nucleic Acids Res* **37**, 4587-4602, doi:10.1093/nar/gkp425 (2009).

7 Westfall, S. D. *et al.* Identification of oxygen-sensitive transcriptional programs in human embryonic stem cells. *Stem Cells Dev* **17**, 869-881, doi:10.1089/scd.2007.0240 (2008).

8 Lange, M. *et al.* CellRank for directed single-cell fate mapping. *Nat Methods* **19**, 159-170, doi:10.1038/s41592-021-01346-6 (2022).

9 Bergen, V., Lange, M., Peidli, S., Wolf, F. A. & Theis, F. J. Generalizing RNA velocity to transient cell states through dynamical modeling. *Nat Biotechnol* **38**, 1408-1414, doi:10.1038/s41587-020-0591-3 (2020).
